# Plasma Proteins Associated with Chronic Histopathologic Lesions on Kidney Biopsy

**DOI:** 10.1681/ASN.0000000000000358

**Published:** 2024-04-24

**Authors:** Taesoo Kim, Aditya L. Surapaneni, Insa M. Schmidt, Michael T. Eadon, Sahir Kalim, Anand Srivastava, Ragnar Palsson, Isaac E. Stillman, Jeffrey B. Hodgin, Rajasree Menon, Edgar A. Otto, Josef Coresh, Morgan E. Grams, Sushrut S. Waikar, Eugene P. Rhee

**Affiliations:** 1 Division of Nephrology, Department of Medicine, Massachusetts General Hospital, Boston, Massachusetts; 2 Department of Medicine, New York University Grossman School of Medicine, New York, New York; 3 Section of Nephrology, Department of Medicine, Boston University Chobanian and Avedisian School of Medicine and Boston Medical Center, Boston, Massachusetts; 4 Department of Medicine, Indiana University School of Medicine, Indianapolis, Indiana; 5 Division of Nephrology, University of Illinois Chicago, Chicago, Illinois; 6 Department of Pathology, Icahn School of Medicine at Mount Sinai, New York, New York; 7 Department of Pathology, University of Michigan, Ann Arbor, Michigan; 8 Department of Computational Medicine and Bioinformatics, University of Michigan, Ann Arbor, Michigan; 9 Division of Nephrology, Department of Internal Medicine, University of Michigan, Ann Arbor, Michigan; 10 Departments of Population Health and Medicine, New York University Grossman School of Medicine, New York, New York; 11 Endocrine Unit, Department of Medicine, Massachusetts General Hospital, Boston, Massachusetts

**Keywords:** arteriosclerosis, CKD, fibrosis, gene expression, glomerulosclerosis, histopathology, interstitial fibrosis, kidney disease, podocyte, glomerular diseases

## Abstract

**Key Points:**

- Proteomic profiling identified 35 blood proteins associated with chronic histopathologic lesions in the kidney.
- Testican-2 was expressed in the glomerulus, released by the kidney into circulation, and inversely associated with glomerulosclerosis severity.
- NELL1 was expressed in tubular epithelial cells, released by the kidney into circulation, and inversely associated with interstitial fibrosis and tubular atrophy severity.

**Background:**

The severity of chronic histopathologic lesions on kidney biopsy is independently associated with higher risk of progressive CKD. Because kidney biopsies are invasive, identification of blood markers that report on underlying kidney histopathology has the potential to enhance CKD care.

**Methods:**

We examined the association between 6592 plasma protein levels measured by aptamers and the severity of interstitial fibrosis and tubular atrophy (IFTA), glomerulosclerosis, arteriolar sclerosis, and arterial sclerosis among 434 participants of the Boston Kidney Biopsy Cohort. For proteins significantly associated with at least one histologic lesion, we assessed renal arteriovenous protein gradients among 21 individuals who had undergone invasive catheterization and assessed the expression of the cognate gene among 47 individuals with single-cell RNA sequencing data in the Kidney Precision Medicine Project.

**Results:**

In models adjusted for eGFR, proteinuria, and demographic factors, we identified 35 proteins associated with one or more chronic histologic lesions, including 20 specific for IFTA, eight specific for glomerulosclerosis, and one specific for arteriolar sclerosis. In general, higher levels of these proteins were associated with more severe histologic score and lower eGFR. Exceptions included testican-2 and NELL1, which were associated with less glomerulosclerosis and IFTA, respectively, and higher eGFR; notably, both of these proteins demonstrated significantly higher levels from artery to renal vein, demonstrating net kidney release. In the Kidney Precision Medicine Project, 13 of the 35 protein hits had cognate gene expression enriched in one or more cell types in the kidney, including podocyte expression of select glomerulosclerosis markers (including testican-2) and tubular expression of several IFTA markers (including NELL1).

**Conclusions:**

Proteomic analysis identified circulating proteins associated with chronic histopathologic lesions, some of which had concordant site-specific expression within the kidney.

## Visual Abstract

**Figure:**
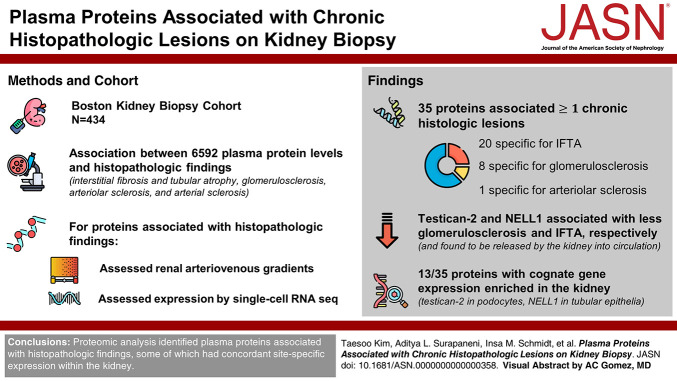

## Introduction

CKD is associated with a range of histopathologic lesions. For example, interstitial fibrosis and tubular atrophy (IFTA) has long been recognized as an important prognostic indicator.^1,2^ Recently, an analysis of individuals who had undergone native kidney biopsy in the Boston Kidney Biopsy Cohort (BKBC) showed that severity of IFTA, global glomerulosclerosis, arteriolar sclerosis, and arterial sclerosis are all independently associated with higher risk of progressive CKD, even after adjusting for eGFR, proteinuria, and clinicopathologic diagnosis.^3^

Current blood markers of kidney function, including creatinine and cystatin C, permit calculation of eGFR and inform risk of CKD progression but do not provide information on underlying kidney histopathology. Because kidney biopsies are invasive and associated with procedural risks,^4^ identification of blood markers that report on underlying kidney histopathology has the potential to enhance CKD care. Previous studies have assessed a limited number of biomarkers in relation to underlying histopathologic lesions.^5,6^ Proteomic approaches now enable high-throughput analysis of thousands of circulating proteins in large cohorts and have been used to identify numerous proteins associated with eGFR and subsequent risk of CKD progression.^7,8^ To what extent these or other proteins are associated with specific kidney pathologic lesions warrants further investigation.

In this study, we examined the association between 6592 circulating plasma protein levels and the severity of IFTA, glomerulosclerosis, arteriolar sclerosis, and arterial sclerosis in the BKBC. Applying a stringent threshold adjusted for multiple hypotheses testing, we considered proteins significantly associated with at least one of these chronic histologic lesions. For these proteins, we assessed levels in renal arteriovenous samples to highlight proteins that may be released by the kidney into circulation. In addition, we sought to corroborate specificity for individual histologic lesions by examining the kidney expression of the corresponding genes using single-cell sequencing data generated by the Kidney Precision Medicine Project (KPMP).

## Methods

### Boston Kidney Biopsy Cohort (BKBC)

The BKBC is a prospective cohort study of adults age 18 years or older undergoing native kidney biopsy at three tertiary care centers in Boston, MA: Beth Israel Deaconess Medical Center, Brigham and Women's Hospital, and Massachusetts General Hospital.^3^ Participants underwent native kidney biopsy between September 2006 and October 2018. Biopsies were performed for clinical indications. Exclusion criteria included the inability to provide written consent, severe anemia, pregnancy, and enrollment in competing studies. A total of 445 participants with available proteomic measurements were included in this study.

### Renal Arteriovenous Sampling

This cohort has been described previously.^9,10^ In brief, patients referred to the Massachusetts General Hospital Cardiac Catheterization Laboratory for right and left heart catheterization were recruited and underwent blood sampling from the renal vein and the abdominal aorta at the level of the renal arteries before coronary artery catheterization (and administration of iodinated contrast medium). All participants were fasting at the time of their procedure.

### Sample Collection and Proteomic Profiling

In BKBC, plasma samples were collected on the day of biopsy for all participants, and aliquots were immediately stored at −80°C. Proteins were assayed using the SOMAScan proteomic platform (SomaScan Assay v4.1).^11^ In addition to plasma samples from the 445 BKBC participants, we analyzed 12 quality control samples and eight blind duplicates. Plasma proteins levels reported in relative fluorescence units were log2-transformed and winsorized at 5 SD from the mean.^12^ Those with mean coefficients of variance >0.5 were excluded. Of 7289 proteins assayed, a total of 6592 proteins passed quality control metrics and were included in the analysis. Principal components were calculated based on the remaining protein levels. Samples that were flagged by SOMAScan or with any of the first ten principal component values beyond 5 SD from the mean were excluded. Of the 445 analyzed BKBC samples, a total of 434 samples met these quality control tests and were included in subsequent analyses.

For the renal arteriovenous sample set, 42 blood samples from 21 individuals (arterial and venous for each individual) were assayed using the SOMAScan proteomic platform (SomaScan Assay v5.0). All 42 samples passed quality control and were included in the analysis.

### Chronic Histopathologic Scores in BKBC

The severity of each of four categories on kidney biopsy—IFTA, glomerulosclerosis, arteriolar sclerosis, and arterial sclerosis—were scored. Two separate kidney pathologists reviewed the biopsies under light microscopy and provided semiquantitative scores for each of these categories, except for the glomerulosclerosis score, which was taken from the official biopsy report as the percentage of sclerosed (global or segmental) glomeruli over total glomeruli. Glomerulosclerosis was scored as 1 for <10% sclerosed glomeruli; 2 for 11%–25% sclerosed glomeruli; 3 for 26%–50% sclerosed glomeruli; and 4 for >50% for sclerosed glomeruli. IFTA, arteriolar sclerosis, and arterial sclerosis were graded on a semiquantitative scale during the study sessions for a score between 1 and 5 for IFTA and 1 and 4 for arteriolar and arterial sclerosis.

The pathologists jointly reviewed each biopsy to resolve any disagreement and reach consensus. In a previous publication, we have shown that the weighted kappa statistic (95% confidence interval [CI]) from 26 randomly selected biopsies for repeat review months after the initial scoring were 0.72 (95% CI, 0.52 to 0.93) for IFTA, 0.66 (95% CI, 0.44 to 0.87) for arteriolar sclerosis, and 0.64 (95% CI, 0.41 to 0.87) for arterial sclerosis.^3^ Scores were missing for four participants with IFTA, one participant with glomerulosclerosis, four participants with arteriolar sclerosis, and six participants with arterial sclerosis.

### Covariate Measurements in BKBC

Participants' medical history and medication lists were collected at the time of kidney biopsy. Demographic data including age, sex, and ethnicity were extracted from the electronic medical records. Serum creatinine levels and spot proteinuria measurements, either urine protein-creatinine ratio (UPCR) or urine albumin-creatinine ratio (UACR), from the day of biopsy were obtained from the electronic medical record. If both UPCR and UACR were available, UACR was used. If only UPCR was available, predicted UACR was calculated using conversion equation: exp (5.2659+0.2934×log (min (PCR/50, 1))+1.5643×log (max(min(PCR/500, 1), 0.1))+1.1109×log (max (PCR/500, 1))−0.0773×(if female)+0.0797×(if diabetic)+0.1265×(if hypertensive)).^13^ If the participant did not have either of these values, we measured UACR from the urine sample collected on the day of kidney biopsy. eGFR was calculated using the Chronic Kidney Disease Epidemiology Collaboration 2021 equation based on creatinine.^14^

### Statistical Analyses

For the primary analysis in BKBC, we examined the association between each plasma protein level (log_2_-transformed) and the severity of each individual chronic histopathologic lesion in linear regression models adjusted for age, sex, ethnicity, eGFR, and proteinuria. Sex and race were treated as categorical variables within the linear regression models. In a secondary analysis, the models were adjusted for age, sex, ethnicity, and eGFR, but not albuminuria. The *β* coefficients in the primary analysis estimate the increase in the protein level (log_2_-transformed) for every point greater histology severity. The threshold for statistical significance was Bonferroni adjusted for 6592 proteins and four histopathologic lesions (*P* < 0.05/(6592×4)=1.9×10^−6^ or −log_10_*P* > 5.7). Because no significant protein associations were identified for arterial sclerosis, only results for the analyses of IFTA, glomerulosclerosis, and arteriolar sclerosis are shown. We also examined Spearman correlation coefficients between all 6592 protein levels and eGFR or proteinuria at the time of the biopsy. Normally distributed covariates were presented as mean (SD), and skewed covariates were presented as median (interquartile range).

For the proteins identified in our primary analysis, we examined renal arteriovenous gradients to identify proteins that may undergo net release by the kidney. The arteriovenous gradient was estimated using the ratio of venous-to-arterial protein levels (V/A ratio). Statistical significance was set at *P* < 0.05 in paired, two-tailed *t* tests.

### Genome-Wide Association Study Look-Ups

For the proteins identified in the primary analysis, we searched the literature for established cis-protein quantitative trait loci (cis-pQTLs). Here, we summarize results from the two largest genome-wide association studies that used the same SomaScan Assay v4 used in BKBC.^15,16^

### Protein Enrichment Localization from Single-Cell RNA Expression

The KPMP is a multicenter prospective cohort study of individuals with AKI or CKD who underwent kidney biopsy.^17^ We accessed publicly available single-cell RNA sequencing data generated on kidney tissue obtained from 47 KPMP participants (20 reference, 12 AKI, 15 CKD) in atlas version 1.^18^ For each gene, these analyses report whether gene expression is enriched within specific kidney cell types relative to all other cell types in the kidney; for cell types with higher expression of a given gene, both the % of cells of that cell type demonstrating expression and the *P* value for enrichment are shown.

For the proteins significantly associated with at least one histopathologic lesion in BKBC, we assessed for significant enrichment of the cognate gene within glomerular, tubular, or vascular/endothelial cell types in the kidney. Glomerular cells include podocytes, parietal epithelial cells (PECs), and mesangial cells (MCs). Tubular cells include proximal tubule epithelial segment 1/segment 2 cells, proximal tubule epithelial segment 3 cells, descending thin limb type 1 cells, medullary thick ascending limb cells, cortical thick ascending limb (C-TAL) cells, distal convoluted tubule type 1 cells, connecting tubule (CNT) cells, CNT intercalated type A (IC-A) cells, CNT principal cells (CNT-PC), IC-A cells, intercalated type B cells, and principal cells (PC). Vascular/endothelial cells include afferent/efferent arteriole endothelial cells, glomerular capillary endothelial cells, vascular smooth muscle cells/pericytes, peritubular capillary endothelial cells, and lymphatic endothelial cells. Other miscellaneous cells include renin-positive juxtaglomerular granular cells, fibroblasts, and myofibroblasts (MyoF).

### Study Oversight

The Partners Human Research Committee approved the BKBC and renal arteriovenous sampling study protocols, which were conducted according to the principles of the Declaration of Helsinki. All participants provided written informed consent. Human samples collected as part of the KPMP consortium (https://KPMP.org) were obtained with informed consent according to the principles of the Declaration of Helsinki and approved by University of Washington Institutional Review Board (20190213).

## Results

### BKBC Study Population Characteristics

Baseline characteristics of the 434 BKBC participants are shown in Table 1 and Supplemental Table 1. The mean age was 54 (16) years, and 206 (48%) were women. The mean serum creatinine was 2.2 (1.9) mg/dl, and the corresponding mean eGFR was 51 (33) ml/min per 1.73 m^2^. The median albuminuria at the time of biopsy was 1.0 (0.2–3.0) g/g creatinine. A total of 256 (59%) participants had a diagnosis of hypertension and 105 (24%) had a diagnosis of diabetes mellitus type 2. The most common indication for kidney biopsy was proteinuria (231, 53%), followed by AKI/abnormal eGFR (178, 41%) and hematuria (107, 25%). The most common primary diagnoses on biopsy were diabetic nephropathy (68, 16%), IgA nephropathy (61, 14%), membranous nephropathy (39, 9%), secondary FSGS (34, 8%), vascular sclerosis (34, 8%), and advanced chronic changes (29, 7%).

**Table 1:** Baseline characteristics of the Boston Kidney Biopsy Cohort

**Baseline demographic characteristics**	**No.**
*N*	434
Age, yr (SD)	54 (16)
Women	206 (48)
Race	
*Asian*	28 (7)
*Black*	87 (20)
*Other*	10 (2)
*Unknown*	21 (5)
*White*	287 (66)
Hispanic	40 (9)
Hypertension	256 (59)
Diabetes mellitus type 1	21 (5)
Diabetes mellitus type 2	105 (24)
UACR, g/g (IQR)	1.0 (0.2–3.0)
eGFR, ml/min per 1.73 m^2^ (SD)	50.9 (33.2)
Serum creatinine, mg/dl (SD)	2.2 (1.9)
**Reason for kidney biopsy^a^**	
Proteinuria	231 (53)
Hematuria	107 (25)
Nephrotic syndrome	50 (12)
Nephritic syndrome	9 (2)
AKI/abnormal eGFR	178 (41)
Unknown CKD/abnormal eGFR	80 (18)
Acute interstitial nephritis	3 (1)
Other	39 (9)
**Primary diagnosis on biopsy**	
Diabetic nephropathy	68 (16)
IgA nephropathy	61 (14)
Membranous nephropathy	39 (9)
Secondary FSGS	34 (8)
Vascular sclerosis	34 (8)
Advanced chronic changes	29 (7)
ANCA vasculitis	21 (5)
Acute tubular necrosis	19 (4)
Thrombotic microangiopathies	16 (4)
Thin basement membrane disease	15 (4)
FSGS	14 (3)
Acute interstitial nephritis	13 (3)
Immune complex GN	13 (3)
Normal	11 (3)
Collapsing GN	8 (2)
Chronic active interstitial nephritis	7 (2)
Oxalate nephropathy	6 (1)
Others^b^	<1%

Data are presented as count with frequency no. (%) for categorical variables and mean±SD and median (interquartile range) for continuous variables. IQR, interquartile range; UACR, urine albumin-creatinine ratio.

a Reasons for kidney biopsy were not mutually exclusive.

b Others: diagnosis with frequency <1%. See Supplemental Table 1 for full list.

### Distribution of Chronic Histopathologic Lesions in BKBC

**Figure 1:**
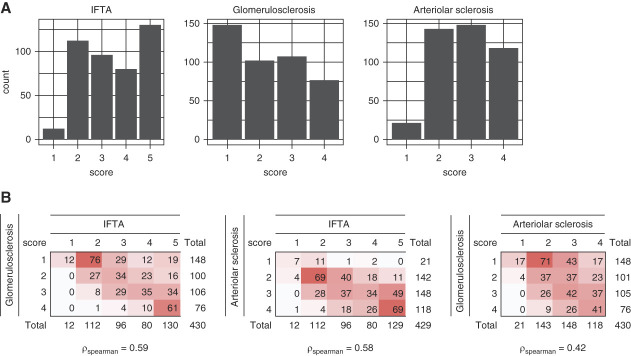
**Histology severity scores in BKBC.** (A) Histology score distributions for IFTA, glomerulosclerosis, and arteriolar sclerosis. Count represents number of participants in each score category. (B) Pair-wise histology score distributions with overlaying heatmap. Darker red represents higher number of participants in the corresponding score categories. BKBC, Boston Kidney Biopsy Cohort; IFTA, interstitial fibrosis and tubular atrophy.

Histology scores for IFTA, glomerulosclerosis, and arteriolar sclerosis were broadly distributed (Figure 1A). The median severity scores were 3 (2–5) for IFTA, 2 (1–3) for glomerulosclerosis, and 3 (2–4) for arteriolar sclerosis. Pair-wise score distributions of the three histopathologic lesions and the corresponding spearman correlations are shown in Figure 1B. Although these pair-wise comparisons demonstrate appreciable correlations in lesion severity, they also highlight areas of discordance. For example, of the 148 individuals with the lowest severity score for glomerulosclerosis, 31 had IFTA scores of 4 or 5 (on a scale from 1 to 5).

### Proteins Associated with Chronic Histopathologic Lesions in BKBC

**Figure 2:**
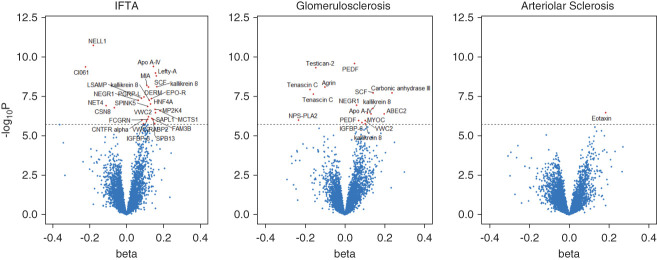
**Proteomic associations with chronic histopathologic lesions in BKBC.** Volcano plots of proteins associated with chronic histologic lesions in BKBC. *β* coefficients and associated log-transformed *P* values for association of 6592 proteins with histologic severity of IFTA (left), glomerulosclerosis (middle), and arteriolar sclerosis (right). Each dot represents a protein, and proteins meeting the threshold for statistical significance (*P* < 1.90×10^−6^), above the dashed line, are labeled.

Proteomic associations with each chronic histopathologic lesion by linear regression are shown as volcano plots in Figure 2. In general, most significantly associated proteins had positive *β* estimates, including erythropoietin receptor (EPO-R, *β* estimate 0.14, −log *P* value 7.4) and hepatocyte NF 4-*α* (HNF4A, *β* estimate 0.13, −log *P* value 7.0) for IFTA and pigment epithelium-derived factor (PEDF) for glomerulosclerosis (*β* estimate 0.05, −log *P* value 9.6); that is, higher protein levels were associated with more severe findings on biopsy. Several proteins, however, had negative *β* estimates, including NELL1 (protein kinase C-binding protein NELL1) for IFTA (*β* estimate −0.18, −log *P*-value 11) and testican-2 for glomerulosclerosis (*β* estimate −0.14, −log *P*-value 9.3).

The proteins significantly associated with at least one chronic histopathologic lesion are shown in Table 2; corresponding box plots for each significant protein association with a histopathologic lesion are shown in Supplemental Figures 1–4. The results of the primary analysis for all proteins are shown in Supplemental Table 2. For four proteins with significant associations, the proteins were detected by two distinct aptamers (all aptamer IDs are provided in Supplemental Table 2).

**Table 2:** Proteins significantly associated with interstitial fibrosis and tubular atrophy, glomerulosclerosis, or arteriolar sclerosis

Protein	Gene	IFTA		Glomerulosclerosis		Arteriolar Sclerosis	
		*β*	−log_10_*P*	*β*	−log_10_*P*	*β*	−log_10_*P*
NELL1 (protein kinase C-binding protein NELL1)	*NELL1*	−0.18	11	—	—	—	—
PEDF	*SERPINF1*	—	—	0.05	9.6	—	—
Apo A-IV	*APOA4*	0.14	9.4	0.11	6.4	—	—
CI061 (protein FAM189A2)	*FAM189A2*	−0.22	9.4	—	—	—	—
Testican-2	*SPOCK2*	—	—	−0.14	9.3	—	—
Lefty-A (left-right determination factor 2)	*LEFTY2*	0.16	9.0	—	—	—	—
SCF	*KITLG*	0.16	8.8	0.14	7.7	—	—
MIA (melanoma-derived growth regulatory protein)	*MIA*	0.11	8.2	—	—	—	—
Kallikrein 8	*KLK8*	0.16	8.1	0.14	6.7	—	—
DERM (dermatopontin)	*DPT*	0.12	8.1	—	—	—	—
Agrin	*AGRN*	—	—	−0.10	8.1	—	—
Tenascin C	*TNC*	—	—	−0.18	7.9	—	—
Carbonic anhydrase III	*CA3*	—	—	0.24	7.7	—	—
PGRP-L (N-acetylmuramoyl-L-alanine amidase)	*PGLYRP2*	0.09	7.4	—	—	—	—
LSAMP (limbic system–associated membrane protein)	*LSAMP*	0.08	7.4	—	—	—	—
EPO-R	*EPOR*	0.14	7.4	—	—	—	—
NEGR1	*NEGR1*	0.06	7.3	0.06	6.9	—	—
HNF4A	*HNF4A*	0.13	7.0	—	—	—	—
NET4 (Netrin-4)	*NTN4*	−0.11	6.9	—	—	—	—
SPINK5 (serine protease inhibitor kazal-type 5)	*SPINK5*	0.11	6.9	—	—	—	—
CSN8 (COP9 signalosome complex subunit 8)	*COPS8*	−0.07	6.8	—	—	—	—
MAP2K4 (dual specificity mitogen-activated protein kinase kinase 4)	*MAP2K4*	0.16	6.7	—	—	—	—
MCTS1 (malignant T-cell–amplified sequence 1)	*MCTS1*	0.18	6.6	—	—	—	—
SAPL1 (proactivator polypeptide-like 1)	*PSAPL1*	0.16	6.5	—	—	—	—
Eotaxin	*CCL11*	—	—	—	—	0.18	6.5
ABEC2 (C->U-editing enzyme APOBEC-2)	*APOBEC2*	—	—	0.20	6.4	—	—
MYOC	*MYOC*	—	—	0.13	6.4	—	—
FAM3B (protein FAM3B)	*FAM3B*	0.14	6.1	—	—	—	—
FCGRN (IgG receptor FcRn large subunit p51)	*FCGRT*	0.11	6.0	—	—	—	—
SPB13 (serpin B13)	*SERPINB13*	0.14	6.0	—	—	—	—
CNTFR*α*	*CNTFR*	0.08	6.0	—	—	—	—
NPS-PLA2 (phospholipase A2, membrane associated)	*PLA2G2A*	—	—	−0.23	6.0	—	—
VWC2 (brorin)	*VWC2*	0.11	5.9	0.10	6.0	—	—
IGFBP-6	*IGFBP6*	0.09	5.8	0.09	5.8	—	—
RABP2 (cellular)	*CRABP2*	0.15	5.8	—	—	—	—

Linear regression models were adjusted for eGFR, proteinuria, and demographic factors (age, sex, and race) to yield *β* estimates. *β*-estimate and log_10_-transformed *P* value pairs for associations significant at the Bonferroni adjusted threshold (*P* < 1.9×10^−6^ or −log_10_*P* > 5.7) are shown. Proteins are ordered on the basis of highest log_10_-transformed *P* value for any lesion. Apo A-IV, apolipoprotein A-IV; CNTFR*α*, ciliary neurotrophic factor receptor subunit *α;* EPO-R, erythropoietin receptor; HNF4A, hepatocyte NF 4-*α;* IFTA, interstitial fibrosis and tubular atrophy; IGFBP-6, insulin-like growth factor-binding protein 6; MYOC, myocilin; NEGR1, neuronal growth regulator 1; PEDF, pigment epithelium–derived factor; RABP2, retinoic acid–binding protein 2; SCF, stem cell factor.

**Figure 3:**
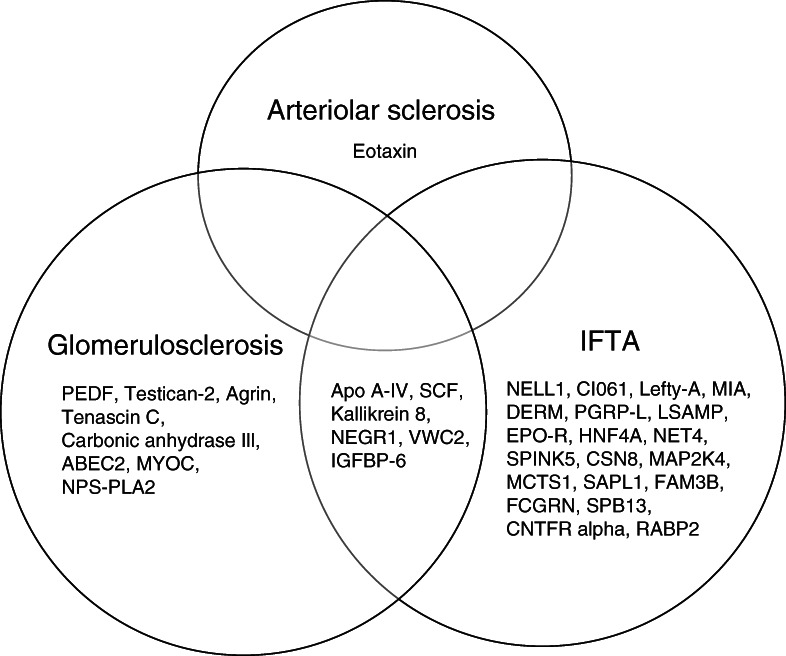
**Specificity of protein associations with chronic histopathologic lesions in BKBC.** Venn diagram showing 35 significant proteins and their association with one or more chronic histopathologic lesions in BKBC. Within each field, proteins are listed from highest to lowest log-transformed *P* value. Apo A-IV, apolipoprotein A-IV; FCGRN, IgG receptor FcRn large subunit p51; IGFBP-6, insulin-like growth factor-binding protein 6; MYOC, myocilin; NEGR1, neuronal growth regulator 1; PEDF, pigment epithelium–derived factor; SCF, stem cell factor.

**Figure 4:**
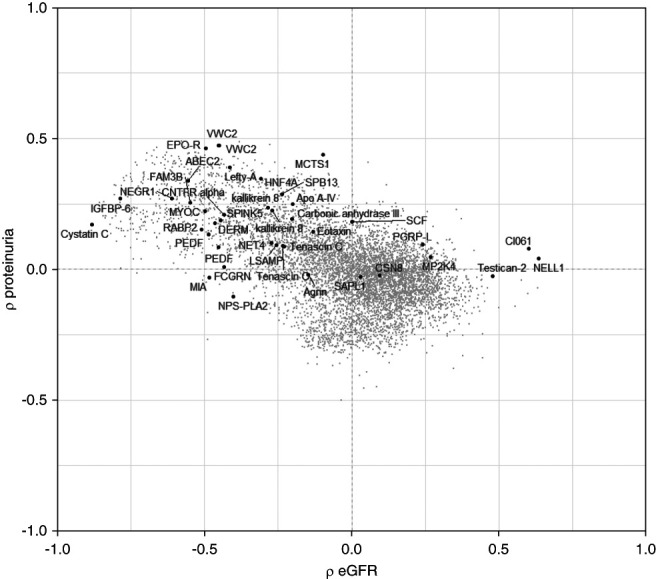
**Protein associations with eGFR and proteinuria in BKBC.** Protein correlations with eGFR and proteinuria. Each protein is plotted based on their spearman correlation coefficient with eGFR (*x* axis) and proteinuria (*y* axis). Proteins with a significant association with one or more chronic histologic lesions, along with cystatin C, are colored black and labeled; all other proteins are colored gray.

Of the 35 unique proteins, 20 proteins were associated with IFTA only, eight were associated with glomerulosclerosis only, and one was associated with arteriolar sclerosis only. Six proteins were associated with both IFTA and glomerulosclerosis, as shown in Figure 3. In secondary analyses that did not adjust for albuminuria, only one of the 35 associations was no longer significant (FCGRN, IgG receptor FcRn large subunit p51, and IFTA); results of the secondary analysis for all proteins are shown in Supplemental Table 3. The majority of the 35 proteins with significant histopathologic associations had a negative cross-sectional correlation with eGFR and positive cross-sectional correlation with proteinuria (Figure 4). Again, notable exceptions included NELL1 (Spearman correlation estimate with eGFR of 0.63) and testican-2 (Spearman correlation estimate with eGFR of 0.48).

### Identification of cis-pQTLs in Genome Wide Association Studies

Notably, cis-pQTLs for 25 of the 35 proteins with significant histopathologic associations have been identified, supporting aptamer specificity (Supplemental Table 4). Of these 25 cis-pQTLs, 13 have also been shown to be in high linkage disequilibrium with a cis-expression quantitative trait loci, providing evidence that tissue gene expression and subsequent blood appearance of these proteins are correlated.

### Renal Arteriovenous Gradients for Chronic Histopathology–Associated Proteins

**Figure 5:**
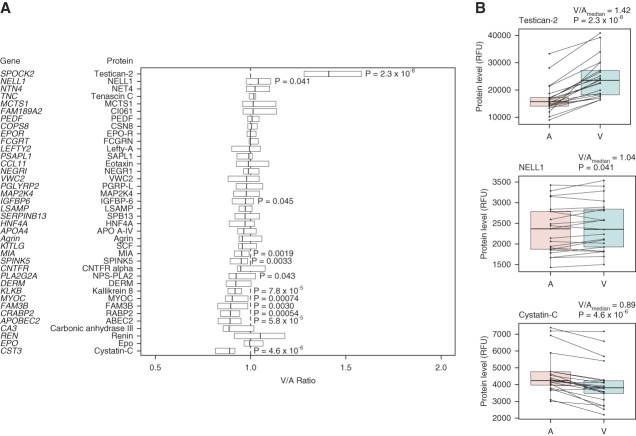
**Renal arteriovenous gradients for proteins associated with chronic histopathologic lesions.** (A) Box plots of venous to arterial protein level (V/A) ratios, with each box denoting median and (IQR). Dashed line represents V/A ratio of 1. *P* values < 0.05 are shown next to corresponding protein box plot. (B) Paired arterial (A) and venous (V) levels for testican-2, NELL1, and cystatin C in all individuals. IQR, interquartile range.

Baseline characteristics of the individuals who underwent renal arteriovenous sampling are shown in Supplemental Table 5. Renal arteriovenous gradients for the 35 proteins significantly associated with chronic histopathologic lesions are shown in Figure 5A along with results for cystatin C, renin, and erythropoietin (Epo). As shown, several proteins showed median venous to arterial (V/A) ratios above 1, although only the artery to vein increases for testican-2 and NELL1 reached statistical significance; testican-2 increased from A to V in all individuals (median V/A=1.42, *P* = 2.3×10^−8^), whereas NELL1 increased from A to V in 14 individuals (median V/A=1.04, *P* = 0.041) (Figure 5B). A significant artery to vein decrease (V/A<1) was noted for ten proteins and cystatin C (median V/A=0.89, *P* = 4.6×10^−6^).

### Kidney Gene Expression for Chronic Histopathology–Associated Proteins in KPMP

**Figure 6:**
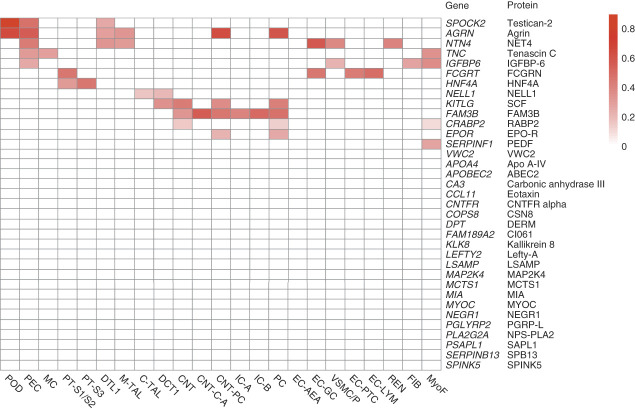
**Kidney expression of genes encoding proteins associated with chronic histopathologic lesions.** Heatmap of KPMP single-cell expression for genes encoding the 35 proteins significantly associated with chronic histopathologic lesions in BKBC. Genes are in rows and kidney cell types are in columns. For each gene, red cells signify cell types with significantly enhanced expression of that gene compared with all other cell types (darker red corresponds to higher % cell expression); 22 genes demonstrated no significant enrichment in any cell type. Glomerular cells include POD, PEC, and MC. Tubular cells include PT-S1/S2 cells, PT-S3 cells, DTL1 cells, M-TAL cells, C-TAL cells, DCT1 cells, CNT cells, CNT-IC-A cells, CNT-PC, IC-A cells, IC-B cells, and PC. Vascular/endothelial cells include EC-AEA, EC-GC, VSMC/P, EC-PTC, and EC-LYM. Other miscellaneous cells are REN, FIB, and MyoF. CNT, connecting tubule; CNT-IC-A, CNT intercalated type A; CNT-PC, CNT principal cell; C-TAL, cortical thick ascending limb; DCT1, distal convoluted tubule type 1; DTL1, descending thin limb type 1; EC-AEA, afferent/efferent arteriole endothelial cells; EC-GC, glomerular capillary endothelial cells; EC-LYM, lymphatic endothelial cells; EC-PTC, peritubular capillary endothelial cells; FIB, fibroblast; IC-A, intercalated type A; IC-B, intercalated type B; KPMP, Kidney Precision Medicine Project; MC, mesangial cell; M-TAL, medullary thick ascending limb; MyoF, myofibroblasts; PC, principal cell; PEC, parietal epithelial cell; POD, podocytes; PT-S1/S2, proximal tubule epithelial segment 1/segment 2; PT-S3, proximal tubule epithelial segment 3; REN, renin-positive juxtaglomerular granular cells; VSMC/P, vascular smooth muscle cells/pericytes.

For the 35 proteins significantly associated with chronic histopathologic lesions in BKBC, we assessed whether expression of the cognate gene was enriched in specific kidney cells using single-cell RNA expression data from KPMP (Figure 6). For 22 proteins, including those associated with arteriolar sclerosis, the corresponding genes demonstrated no significant enrichment of expression in specific kidney cells. For the remaining 13 proteins, the corresponding genes demonstrated significant enrichment in tubular cells, glomerular cells, or both in KPMP samples.

Interestingly, genes corresponding to the proteins associated with IFTA in BKBC were distributed heterogeneously across the tubular epithelium. For example, *HNF4A* (encodes HNF4A) was enriched in proximal tubule epithelial segment 1/segment 2 and proximal tubule epithelial segment 3 cells (32% and 46%, respectively), *NELL1* was enriched in C-TAL and distal convoluted tubule type 1 cells (17% and 20%, respectively), and *EPOR* (encodes EPO-R) was enriched in CNT-PC and PC (22% and 26%, respectively). *FAM3B* (encodes protein FAM3B) was enriched in CNT cells (34%), CNT IC-A cells (57%), CNT-PCs (45%), IC-A cells (41%), intercalated type B cells (52%), and PCs (47%).

For several proteins significantly associated with glomerulosclerosis in BKBC, the corresponding genes were highly expressed in glomerular cells in KPMP. For example, *SPOCK2* (encodes testican-2) demonstrated higher expression in podocytes and PECs (89% and 48%, respectively). *AGRN* had greater expression in podocytes and PECs (66% and 57%, respectively), as well as in several tubular cells (30% descending thin limb type 1 cells, 34% medullary thick ascending limb cells, 63% CNT-PC, and 59% PCs). *TNC* was significantly higher in PECs and MCs (31% and 30%, respectively), as well as MyoF (34%). *SERPINF1* (encodes PEDF) was significantly greater in MyoF (28%).

Supplemental Figure 5 shows representative uniform manifold approximation and projection demonstrating single-cell RNA expression in the kidney for *NELL1*, *HNF4A*, *SPOCK2*, and *TNC*.

## Discussion

Using a proteomic approach, this study identifies plasma proteins significantly associated with the severity of chronic histopathologic lesions on kidney biopsy. Many of the proteins are associated with a single histopathologic lesion, despite the moderate intercorrelation of severity scores for IFTA, glomerulosclerosis, and arteriolar sclerosis. Renal arteriovenous profiling demonstrates that several of these proteins may be released by the kidney into circulation. Furthermore, the genes encoding several of these proteins demonstrate increased expression in specific cell types across the nephron, for example in tubular epithelial cells or glomerular cells that align with the observed protein-histology associations. Together, these findings nominate select circulating proteins as potential noninvasive measures of underlying kidney histopathology, particularly NELL1 for IFTA and testican-2 for glomerulosclerosis.

Using the same renal arteriovenous sample cohort, but with a more limited proteomic analysis, we have previously shown that testican-2 increases from artery to renal vein.^10^ For this study, we have now used an updated version of the same aptamer-based platform to measure all 35 proteins associated with a chronic histopathologic lesion in BKBC. In addition to recapitulating our previous result with testican-2, we now show that NELL1 also increases significantly from artery to renal vein. Although median V/A values>1 for several other proteins, including HNF4A, EPO-R, tenascin-C, and PEDF, did not reach statistical significance, our results raise the possibility that they can be released by the kidney in some individuals (we note that the A to V changes for renin and Epo were also not statistically significant, but similarly showed A to V increases in some individuals). By contrast, some proteins such as insulin-like growth factor-binding protein 6, stem cell factor, and FAM3B decreased from A to V in most or all samples, making their net release from the kidney unlikely—therefore, we do not discuss these proteins further, even if they were found to be enriched in specific cells within the kidney in KPMP.

As noted, several of the proteins associated with IFTA had increased expression of their cognate genes in distinct segments of the tubular epithelium. Heterozygous mutations in *HNF4A*, enriched in the proximal tubule, are associated with maturity onset diabetes of the young,^19,20^ as well as proximal tubulopathy (Fanconi syndrome).^21,22^ Recent studies have shown that HNF4A, a nuclear hormone receptor, is crucial for the development of proximal tubules in mice.^23,24^ Similarly, human kidney organoids lacking HNF4A show reduced expression of transport-, endocytosis-, and brush border–related genes in the apical lumen of the organoid proximal tubule.^25^ Although most studies to date have underscored a fundamental role for HNF4A in proximal tubule development and differentiation,^26^ our findings identify the protein as a potential marker of tubulointerstitial fibrosis and atrophy. More work is required to determine whether it may play a deleterious or adaptive role in this context.

Like HNF4A, NELL1 is an IFTA-associated protein that has been implicated in human kidney disease. More specifically, NELL1 is a recently discovered target antigen in some cases of membranous nephropathy, with colocalization of NELL1 and IgG along the glomerular basement membrane and anti-NELL1 antibodies in serum.^27,28^ Initially described in cases of idiopathic membranous nephropathy, anti-NELL1 antibodies have subsequently been found in a variety of settings, including malignancy, drugs, infections, autoimmune disease, and after hematopoietic stem cell transplant.^29^ Despite this association with a glomerular disease, we find that *NELL1* expression is enriched in the C-TAL and distal convoluted tubule, and circulating levels of NELL1 are associated with IFTA. In bone, NELL1 deficiency causes skeletal defects with reduced expression of extracellular matrix proteins,^30^ whereas NELL1 expression promotes bone formation by inducing Wnt/*β*-catenin signaling.^31,32^ Transient activation of the Wnt/*β*-catenin system stimulates tissue regeneration after AKI, whereas sustained signaling promotes kidney fibrosis^33^; whether NELL1 expression in the kidney affects this signaling pathway is unknown.

EPO-R, the receptor for erythropoietin, mediates erythroblast proliferation and differentiation. We find that EPO-R levels are associated with IFTA and that its expression is increased in the CNT and PC, but in this case, the literature suggests that elevated EPO-R expression may be adaptive. EPO-R/erythropoietin signaling has been implicated in protection against ischemic injury in various organ systems, including the nervous system, myocardium, skin, liver, and kidney.^34–39^ Furthermore, mice with tubule-specific *Epor* deletion have more severe AKI and more tubulointerstitial fibrosis after ischemia-reperfusion injury.^38^ Therefore, the association observed between EPO-R and IFTA in BKBC may reflect a compensatory, rather than causal, role for erythropoietin signaling in response to chronic tubular injury.

For several proteins associated with glomerulosclerosis, we find higher expression of their cognate genes in the glomerulus. For example, we find higher expression of *SPOCK2* in podocytes and PECs (as well as descending thin limb cells) and higher expression of *TNC* (encodes tenascin C) in PECs and MCs (and MyoF). *SPOCK2* encodes testican-2, a glycoprotein that we have previously shown is podocyte-derived and secreted across the glomerular basement membrane and into circulation.^10^ In addition, we have shown that higher blood testican-2 levels are associated with less eGFR loss over time in population-based cohorts (*n*>3500) and lower risk of incident kidney failure across three large studies (*n*>8000), even after adjusting for baseline eGFR, albuminuria, and other CKD risk factors. ^10,40^ Like testican-2, tenascin C is a glycoprotein that is secreted into the extracellular matrix.^41,42^ It is thought to play a key role in regulating cell proliferation, migration, and angiogenesis in various tissues.^43,44^ In kidney biopsy samples from individuals with diabetic nephropathy and IgA nephropathy, tenascin C expression was greatest in MC, and tenascin C treatment of primary MC increased MC proliferation and matrix expansion.^45,46^

Although our discussion primarily emphasizes proteins whose expression localize to tubular and glomerular cells, we note that this only represents a subset of the proteins significantly associated with chronic histopathologic lesions in BKBC. For example, expression of *SERPNF1*, which encodes the glomerulosclerosis-associated protein PEDF, is significantly higher in MyoF, but not in any other kidney cells. PEDF is a circulating glycoprotein with antiangiogenic and anti-inflammatory properties,^47^ and elevated blood levels have been previously associated with progression of diabetic nephropathy.^48^ In a targeted analysis using a Luminex-based assay, we have previously shown that higher levels of PEDF in both blood and urine are associated with IFTA^49^; in the current aptamer-based analysis, we do observe a nominal association between PEDF and IFTA but one that is significantly weaker than the association observed with glomerulosclerosis.

As shown by the strong rightward skew in the Figure 2 Volcano plots, most significant associations highlight proteins for which higher levels signify more severe chronic histopathologic lesions. Notable exceptions, already discussed herein, include NELL1, testican-2, and tenascin C. In contrast to most of the other significant protein hits, NELL1 and testican-2 levels are also positively associated with eGFR and increase significantly from artery to renal vein. The possibility that a molecule's association with kidney disease severity simply reflects its passive accumulation because of reduced renal clearance is an important consideration in CKD research and one that is not entirely abrogated by statistical adjustment for eGFR. However, this is not applicable to NELL1 and testican-2, with levels that were higher with higher eGFR, thus outlining potential markers of tubular and glomerular health, respectively.

Our study has several notable strengths. These include the large number of proteins assayed, the large patient cohort with kidney histopathology adjudicated by two nephropathologists, the assessment of protein levels in renal arteriovenous samples, and the incorporation of emerging data from the KPMP. Several limitations also warrant mention, including the lack of an independent validation cohort for BKBC, the single time point for proteomic profiling and kidney biopsy per study participant, and the observational nature of our analyses that precludes assignment of causal relationships. In addition, because the correlation between mRNA and protein expression can vary substantially, we look forward to future analyses that will systematically compare these molecular domains in KPMP.

In sum, proteomic analysis identifies circulating proteins associated with chronic histopathologic lesions. We believe our findings can inform future research along several axes. For example, they highlight several proteins with potential mechanistic relevance to IFTA and glomerulosclerosis that warrant further study, including for therapeutic targeting. To this end, we prioritize associations for which the protein-histopathology and cognate gene–kidney cell expression are concordant. In addition, our findings motivate future studies in other patient populations, both to permit replication and to extend generalizability to other contexts where the presence or absence of chronic histopathologic lesions is highly relevant to prognostication and clinical decision making, such as kidney transplantation. Ideally, these studies would include repeated sampling over time and monitor clinical outcomes to permit assessment of whether blood markers of chronic lesions change in tandem with underlying histopathology and whether they can predict the response, or absence of response, to therapy.

## Supplementary Material

SUPPLEMENTARY MATERIAL

## Data Availability

Partial restrictions to the data and/or materials apply. Associations between all proteins and chronic histopathologic lesions in BKBC are available in Supplemental Tables 2 and 3 in an excel format. The underlying individual data cannot be shared publicly due to privacy of individuals that participated in the study. The single-cell RNA dataset generated in the KPMP cohort is publicly available at https://KPMP.org.

## Supplemental Material

Supplemental Table 1. Demographic characteristics of BKBC.

Supplemental Table 2. Protein associations with chronic histologic lesions, adjusted for demographics, eGFR, and proteinuria.

Supplemental Table 3. Protein associations with chronic histologic lesions, adjusted for demographics and eGFR.

Supplemental Table 4. cis-pQTLs and cis-eQTLs for proteins associated with chronic histologic lesions in BKBC.

Supplemental Table 5. Demographic characteristics of the renal arteriovenous sampling cohort.

Supplemental Figure 1. Proteins significantly associated with IFTA.

Supplemental Figure 2. Proteins significantly associated with glomerulosclerosis.

Supplemental Figure 3. Protein significantly associated with arteriolar sclerosis.

Supplemental Figure 4. Proteins significantly associated with >1 chronic histologic lesion.

Supplemental Figure 5. UMAPs for representative genes with significantly increased expression in kidney cells.
