# Supplementary material for: Plasma Proteins Associated with Chronic Histopathologic Lesions on Kidney Biopsy
Source: J Am Soc Nephrol. 2024 Apr 24;35(7):910–22. doi: 10.1681/ASN.0000000000000358 (PMC11230715; doi:10.1681/ASN.0000000000000358)
Supplement: SUPPLEMENTARY MATERIAL [file jasn-35-910-s001.pdf]

ASN American Society of Nephrology
JASN Journal of the American Society of Nephrology
KIDNEY360 Accessing Our World From Every Angle
CJASN Clinical Journal of the American Society of Nephrology

ASN Journal Disclosure Form

As per ASN journal policy, I have disclosed any financial relationship or commitment held by myself and/or my spouse/partner in the past 36 months as included below. I have listed my Current Employer below to indicate there is a relationship requiring disclosure. If no relationship exists, my Current Employer is not listed.

J. Coresh reports the following:
Employer: The Johns Hopkins University, Welch Center for Prevention Epidemiology & Clinical Research; Consultancy: Scientific Advisory Board - Healthy.io and SomaLogic; Ownership Interest: Healthy.io; and Research Funding: National Institute of Health and NKF.

I understand that the information above will be published within the journal article, if accepted, and that failure to comply and/or to accurately and completely report the potential financial conflicts of interest could lead to the following: 1) Prior to publication, article rejection, or 2) Post-publication, sanctions ranging from, but not limited to, issuing a correction, reporting the inaccurate information to the authors' institution, banning authors from submitting work to ASN journals for varying lengths of time, and/or retraction of the published work.

Name: Josef Coresh
Manuscript ID: JASN-2023-001525R1
Manuscript Title: Plasma Proteins Associated with Chronic Histopathologic Lesions on Kidney Biopsy
Date of Completion: March 29, 2024
Disclosure Updated Date: October 30, 2023

ASN American Society of Nephrology | JASN Journal of the American Society of Nephrology | KIDNEY360 Accessing Our World From Every Angle | CJASN Clinical Journal of the American Society of Nephrology

ASN Journal Disclosure Form

As per ASN journal policy, I have disclosed any financial relationship or commitment held by myself and/or my spouse/partner in the past 36 months as included below. I have listed my Current Employer below to indicate there is a relationship requiring disclosure. If no relationship exists, my Current Employer is not listed.

M. Eadon reports the following:
Employer: Indiana University

I understand that the information above will be published within the journal article, if accepted, and that failure to comply and/or to accurately and completely report the potential financial conflicts of interest could lead to the following: 1) Prior to publication, article rejection, or 2) Post-publication, sanctions ranging from, but not limited to, issuing a correction, reporting the inaccurate information to the authors' institution, banning authors from submitting work to ASN journals for varying lengths of time, and/or retraction of the published work.

Name: Michael T. Eadon
Manuscript ID: JASN-2023-001525R1
Manuscript Title: Plasma Proteins Associated with Chronic Histopathologic Lesions on Kidney Biopsy
Date of Completion: March 26, 2024
Disclosure Updated Date: August 27, 2023

ASN Journal Disclosure Form

As per ASN journal policy, I have disclosed any financial relationship or commitment held by myself and/or my spouse/partner in the past 36 months as included below. I have listed my Current Employer below to indicate there is a relationship requiring disclosure. If no relationship exists, my Current Employer is not listed.

M. Grams reports the following:
Employer: New York University; Advisory or Leadership Role: AJKD; ASN Editorial Committee; NKF Scientific Advisory Board; KDIGO Co-Chair, USRDS Scientific Advisory Board; ASN Publication Committee, KRI Scientific Advisory Committee; and Other Interests or Relationships: Grant funding from NKF -- which receives funding from multiple pharmaceutical companies; grant funding from NIH; payment from academic institutions for grand rounds; payment from NephSAP; travel reimbursement from KDIGO and Kidney Research Initiative and CDC, travel reimbursement and stipend from Korean Society of Nephrology and Hong Kong Society of Nephrology.

I understand that the information above will be published within the journal article, if accepted, and that failure to comply and/or to accurately and completely report the potential financial conflicts of interest could lead to the following: 1) Prior to publication, article rejection, or 2) Post-publication, sanctions ranging from, but not limited to, issuing a correction, reporting the inaccurate information to the authors' institution, banning authors from submitting work to ASN journals for varying lengths of time, and/or retraction of the published work.

Name: Morgan Grams
Manuscript ID: JASN-2023-001525R1
Manuscript Title: Plasma Proteins Associated with Chronic Histopathologic Lesions on Kidney Biopsy
Date of Completion: March 14, 2024
Disclosure Updated Date: March 12, 2024

ASN American Society of Nephrology | JASN Journal of the American Society of Nephrology | KIDNEY360 Accessing Our World From Every Angle | CJASN Clinical Journal of the American Society of Nephrology

ASN Journal Disclosure Form

As per ASN journal policy, I have disclosed any financial relationships or commitments I have held in the past 36 months as included below. I have listed my Current Employer below to indicate there is a relationship requiring disclosure. If no relationship exists, my Current Employer is not listed.

J. Hodgin reports the following:
Employer: The University of Michigan; and Research Funding: AstraZeneca; Moderna; Gilead; Janssen; Novo Nordisk; Eli Lilly;.

I understand that the information above will be published within the journal article, if accepted, and that failure to comply and/or to accurately and completely report the potential financial conflicts of interest could lead to the following: 1) Prior to publication, article rejection, or 2) Post-publication, sanctions ranging from, but not limited to, issuing a correction, reporting the inaccurate information to the authors' institution, banning authors from submitting work to ASN journals for varying lengths of time, and/or retraction of the published work.

Name: Jeffrey B. Hodgin
Manuscript ID: JASN-2023-001525R2
Manuscript Title: Plasma Proteins associated with Chronic Histopathologic Lesions on Kidney Biopsy
Date of Completion: April 17, 2024
Disclosure Updated Date: March 28, 2024

ASN American Society of Nephrology JASN Journal of the American Society of Nephrology KIDNEY360 Accessing Our World From Every Angle CJASN Clinical Journal of the American Society of Nephrology

ASN Journal Disclosure Form

As per ASN journal policy, I have disclosed any financial relationship or commitment held by myself and/or my spouse/partner in the past 36 months as included below. I have listed my Current Employer below to indicate there is a relationship requiring disclosure. If no relationship exists, my Current Employer is not listed.

S. Kalim reports the following:
Employer: Massachusetts General Hospital/ Harvard Medical School; Consultancy: Fresenius Kabi; Lediant; Alnylam; Vistera; and Speakers Bureau: Fresenius Kabi.

I understand that the information above will be published within the journal article, if accepted, and that failure to comply and/or to accurately and completely report the potential financial conflicts of interest could lead to the following: 1) Prior to publication, article rejection, or 2) Post-publication, sanctions ranging from, but not limited to, issuing a correction, reporting the inaccurate information to the authors' institution, banning authors from submitting work to ASN journals for varying lengths of time, and/or retraction of the published work.

Name: Sahir Kalim
Manuscript ID: JASN-2023-001525R1
Manuscript Title: Plasma Proteins Associated with Chronic Histopathologic Lesions on Kidney Biopsy
Date of Completion: March 15, 2024
Disclosure Updated Date: March 15, 2024

ASN American Society of Nephrology | JASN Journal of the American Society of Nephrology | KIDNEY360 Accessing Our World From Every Angle | CJASN Clinical Journal of the American Society of Nephrology

ASN Journal Disclosure Form

As per ASN journal policy, I have disclosed any financial relationship or commitment held by myself and/or my spouse/partner in the past 36 months as included below. I have listed my Current Employer below to indicate there is a relationship requiring disclosure. If no relationship exists, my Current Employer is not listed.

T. Kim reports the following:
Employer: Brigham and Women's Hospital/Massachusetts General Hospital

I understand that the information above will be published within the journal article, if accepted, and that failure to comply and/or to accurately and completely report the potential financial conflicts of interest could lead to the following: 1) Prior to publication, article rejection, or 2) Post-publication, sanctions ranging from, but not limited to, issuing a correction, reporting the inaccurate information to the authors' institution, banning authors from submitting work to ASN journals for varying lengths of time, and/or retraction of the published work.

Name: Taesoo Kim
Manuscript ID: JASN-2023-001525R1
Manuscript Title: Plasma Proteins Associated with Chronic Histopathologic Lesions on Kidney Biopsy
Date of Completion: March 14, 2024
Disclosure Updated Date: March 14, 2024

ASN
American Society of Nephrology
JASN
Journal of the American Society of Nephrology
KIDNEY360
Accessing Our World From Every Angle
CJASN
Clinical Journal of the American Society of Nephrology

ASN Journal Disclosure Form

As per ASN journal policy, I have disclosed any financial relationship or commitment held by myself and/or my spouse/partner in the past 36 months as included below. I have listed my Current Employer below to indicate there is a relationship requiring disclosure. If no relationship exists, my Current Employer is not listed.

R. Menon reports the following:
Employer: University of Michigan

I understand that the information above will be published within the journal article, if accepted, and that failure to comply and/or to accurately and completely report the potential financial conflicts of interest could lead to the following: 1) Prior to publication, article rejection, or 2) Post-publication, sanctions ranging from, but not limited to, issuing a correction, reporting the inaccurate information to the authors' institution, banning authors from submitting work to ASN journals for varying lengths of time, and/or retraction of the published work.

Name: Rajasree Menon
Manuscript ID: JASN-2023-001525R1
Manuscript Title: Plasma Proteins Associated with Chronic Histopathologic Lesions on Kidney Biopsy
Date of Completion: March 13, 2024
Disclosure Updated Date: May 15, 2023

ASN American Society of Nephrology JASN Journal of the American Society of Nephrology KIDNEY360 Accessing Our World From Every Angle CJASN Clinical Journal of the American Society of Nephrology

ASN Journal Disclosure Form

As per ASN journal policy, I have disclosed any financial relationship or commitment held by myself and/or my spouse/partner in the past 36 months as included below. I have listed my Current Employer below to indicate there is a relationship requiring disclosure. If no relationship exists, my Current Employer is not listed.

E. Otto reports the following:
Employer: University of Michigan; and Patents or Royalties: Athena Diagnostics.

I understand that the information above will be published within the journal article, if accepted, and that failure to comply and/or to accurately and completely report the potential financial conflicts of interest could lead to the following: 1) Prior to publication, article rejection, or 2) Post-publication, sanctions ranging from, but not limited to, issuing a correction, reporting the inaccurate information to the authors' institution, banning authors from submitting work to ASN journals for varying lengths of time, and/or retraction of the published work.

Name: Edgar A. Otto
Manuscript ID: JASN-2023-001525R1
Manuscript Title: Plasma Proteins Associated with Chronic Histopathologic Lesions on Kidney Biopsy
Date of Completion: March 26, 2024
Disclosure Updated Date: March 26, 2024

ASN American Society of Nephrology
JASN Journal of the American Society of Nephrology
KIDNEY360 Accessing Our World From Every Angle
CJASN Clinical Journal of the American Society of Nephrology

ASN Journal Disclosure Form

As per ASN journal policy, I have disclosed any financial relationship or commitment held by myself and/or my spouse/partner in the past 36 months as included below. I have listed my Current Employer below to indicate there is a relationship requiring disclosure. If no relationship exists, my Current Employer is not listed.

R. Palsson reports the following:
Employer: Massachusetts General Hospital, Boston, MA

I understand that the information above will be published within the journal article, if accepted, and that failure to comply and/or to accurately and completely report the potential financial conflicts of interest could lead to the following: 1) Prior to publication, article rejection, or 2) Post-publication, sanctions ranging from, but not limited to, issuing a correction, reporting the inaccurate information to the authors' institution, banning authors from submitting work to ASN journals for varying lengths of time, and/or retraction of the published work.

Name: Ragnar Palsson
Manuscript ID: JASN-2023-001525R1
Manuscript Title: Plasma Proteins Associated with Chronic Histopathologic Lesions on Kidney Biopsy
Date of Completion: March 15, 2024
Disclosure Updated Date: May 23, 2023

ASN American Society of Nephrology
JASN Journal of the American Society of Nephrology
KIDNEY360 Accessing Our World From Every Angle
CJASN Clinical Journal of the American Society of Nephrology

ASN Journal Disclosure Form

As per ASN journal policy, I have disclosed any financial relationship or commitment held by myself and/or my spouse/partner in the past 36 months as included below. I have listed my Current Employer below to indicate there is a relationship requiring disclosure. If no relationship exists, my Current Employer is not listed.

E. Rhee reports the following:
Employer: Massachusetts General Hospital

I understand that the information above will be published within the journal article, if accepted, and that failure to comply and/or to accurately and completely report the potential financial conflicts of interest could lead to the following: 1) Prior to publication, article rejection, or 2) Post-publication, sanctions ranging from, but not limited to, issuing a correction, reporting the inaccurate information to the authors' institution, banning authors from submitting work to ASN journals for varying lengths of time, and/or retraction of the published work.

Name: Eugene P. Rhee
Manuscript ID: JASN-2023-001525R1
Manuscript Title: Plasma Proteins Associated with Chronic Histopathologic Lesions on Kidney Biopsy
Date of Completion: March 13, 2024
Disclosure Updated Date: March 13, 2024

ASN American Society of Nephrology
JASN Journal of the American Society of Nephrology
KIDNEY360 Accessing Our World From Every Angle
CJASN Clinical Journal of the American Society of Nephrology

ASN Journal Disclosure Form

As per ASN journal policy, I have disclosed any financial relationship or commitment held by myself and/or my spouse/partner in the past 36 months as included below. I have listed my Current Employer below to indicate there is a relationship requiring disclosure. If no relationship exists, my Current Employer is not listed.

I. Schmidt has nothing to disclose.

I understand that the information above will be published within the journal article, if accepted, and that failure to comply and/or to accurately and completely report the potential financial conflicts of interest could lead to the following: 1) Prior to publication, article rejection, or 2) Post-publication, sanctions ranging from, but not limited to, issuing a correction, reporting the inaccurate information to the authors' institution, banning authors from submitting work to ASN journals for varying lengths of time, and/or retraction of the published work.

Name: Insa Marie Schmidt
Manuscript ID: JASN-2023-001525R1
Manuscript Title: Plasma Proteins Associated with Chronic Histopathologic Lesions on Kidney Biopsy
Date of Completion: March 15, 2024
Disclosure Updated Date: February 16, 2024

ASN American Society of Nephrology JASN Journal of the American Society of Nephrology KIDNEY360 Accessing Our World From Every Angle CJASN Clinical Journal of the American Society of Nephrology

ASN Journal Disclosure Form

As per ASN journal policy, I have disclosed any financial relationship or commitment held by myself and/or my spouse/partner in the past 36 months as included below. I have listed my Current Employer below to indicate there is a relationship requiring disclosure. If no relationship exists, my Current Employer is not listed.

A. Srivastava reports the following:
Employer: University of Illinois Chicago; Consultancy: CVS Caremark; and Honoraria: Horizon Therapeutics PLC; AstraZeneca; Bayer; FNIH; University of Chicago; University of Washington; American Diabetes Association.

I understand that the information above will be published within the journal article, if accepted, and that failure to comply and/or to accurately and completely report the potential financial conflicts of interest could lead to the following: 1) Prior to publication, article rejection, or 2) Post-publication, sanctions ranging from, but not limited to, issuing a correction, reporting the inaccurate information to the authors' institution, banning authors from submitting work to ASN journals for varying lengths of time, and/or retraction of the published work.

Name: Anand Srivastava
Manuscript ID: JASN-2023-001525R1
Manuscript Title: Plasma Proteins Associated with Chronic Histopathologic Lesions on Kidney Biopsy
Date of Completion: March 14, 2024
Disclosure Updated Date: March 1, 2024

ASN Journal Disclosure Form

As per ASN journal policy, I have disclosed any financial relationships or commitments I have held in the past 36 months as included below. I have listed my Current Employer below to indicate there is a relationship requiring disclosure. If no relationship exists, my Current Employer is not listed.

I. Stillman reports the following:
Employer: Mount Sinai Hospital; Icahn School of Medicine at Mount Sinai; and Other Interests or Relationships: Legal Consulting Less than 15K per year.

I understand that the information above will be published within the journal article, if accepted, and that failure to comply and/or to accurately and completely report the potential financial conflicts of interest could lead to the following: 1) Prior to publication, article rejection, or 2) Post-publication, sanctions ranging from, but not limited to, issuing a correction, reporting the inaccurate information to the authors' institution, banning authors from submitting work to ASN journals for varying lengths of time, and/or retraction of the published work.

Name: Isaac Ely Stillman
Manuscript ID: JASN-2023-001525R2
Manuscript Title: "Plasma Proteins associated with Chronic Histopathologic Lesions on Kidney Biopsy,"
Date of Completion: April 17, 2024
Disclosure Updated Date: April 17, 2024

ASN American Society of Nephrology
JASN Journal of the American Society of Nephrology
KIDNEY360 Accessing Our World From Every Angle
CJASN Clinical Journal of the American Society of Nephrology

ASN Journal Disclosure Form

As per ASN journal policy, I have disclosed any financial relationship or commitment held by myself and/or my spouse/partner in the past 36 months as included below. I have listed my Current Employer below to indicate there is a relationship requiring disclosure. If no relationship exists, my Current Employer is not listed.

A. Surapaneni reports the following:
Employer: NYU Langone Health

I understand that the information above will be published within the journal article, if accepted, and that failure to comply and/or to accurately and completely report the potential financial conflicts of interest could lead to the following: 1) Prior to publication, article rejection, or 2) Post-publication, sanctions ranging from, but not limited to, issuing a correction, reporting the inaccurate information to the authors' institution, banning authors from submitting work to ASN journals for varying lengths of time, and/or retraction of the published work.

Name: Aditya L. Surapaneni
Manuscript ID: JASN-2023-001525R1
Manuscript Title: Plasma Proteins Associated with Chronic Histopathologic Lesions on Kidney Biopsy
Date of Completion: March 14, 2024
Disclosure Updated Date: May 12, 2023

ASN
American Society of Nephrology
JASN
Journal of the American Society of Nephrology
KIDNEY360
Accessing Our World From Every Angle
CJASN
Clinical Journal of the American Society of Nephrology

ASN Journal Disclosure Form

As per ASN journal policy, I have disclosed any financial relationship or commitment held by myself and/or my spouse/partner in the past 36 months as included below. I have listed my Current Employer below to indicate there is a relationship requiring disclosure. If no relationship exists, my Current Employer is not listed.

S. Waikar reports the following:
Employer: Boston University, Google (spouse); Consultancy: Wolters Kluewer, Bain, BioMarin, Goldfinch, GSK, Ikena, Strataca, Google, CANbridge, NovoNordisk, Ono, PepGen, Sironax, NovoNordisk, Vertex, Mineralys, Motric Bio; Research Funding: Vertex, Pfizer, JNJ, Natera; and Other Interests or Relationships: expert witness for litigation related to dialysis lab testing (Davita), PPIs (Pfizer), PFAO exposure (Dechert), voclosporin patent (Aurinia).

I understand that the information above will be published within the journal article, if accepted, and that failure to comply and/or to accurately and completely report the potential financial conflicts of interest could lead to the following: 1) Prior to publication, article rejection, or 2) Post-publication, sanctions ranging from, but not limited to, issuing a correction, reporting the inaccurate information to the authors' institution, banning authors from submitting work to ASN journals for varying lengths of time, and/or retraction of the published work.

Name: Sushrut S. Waikar
Manuscript ID: JASN-2023-001525R1
Manuscript Title: Plasma Proteins Associated with Chronic Histopathologic Lesions on Kidney Biopsy
Date of Completion: March 26, 2024
Disclosure Updated Date: March 21, 2024
